# Evidence of Australian wild deer exposure to *N. caninum* infection and potential implications for the maintenance of *N. caninum* sylvatic cycle

**DOI:** 10.1186/s12917-023-03712-2

**Published:** 2023-09-13

**Authors:** Jose L. Huaman, Carlo Pacioni, Mark Doyle, David M. Forsyth, Karla J. Helbig, Teresa G. Carvalho

**Affiliations:** 1 Department of Microbiology, Anatomy, Physiology and Pharmacology, School of Agriculture, Biomedicine and Environment, La Trobe University, Melbourne, VIC Australia; 2 Department of Environment, Arthur Rylah Institute for Environmental Research, Land, Water and Planning, Melbourne, VIC Australia; 3 Environmental and Conservation Sciences, Murdoch University, Perth, WA Australia; 4 South East Local Land Services, Bega, NSW Australia; 5 Department of Primary Industries, Vertebrate Pest Research Unit, NSW, Orange Agricultural Institute, Orange, NSW Australia

**Keywords:** Sylvatic cycle, Neosporosis, Deer, Seroprevalence

## Abstract

Infections with the coccidian parasite *Neospora caninum* affect domestic and wild animals worldwide. In Australia, *N. caninum* infections cause considerable losses to the cattle industry with seroprevalence of 8.7% in beef and 10.9% in dairy cattle. Conversely, the role of wild animals, in maintaining the parasite cycle is also unclear. It is possible that native or introduced herbivorous species could be reservoir hosts of *N. caninum* in Australia, but to date, this has not been investigated. We report here the first large-scale screening of *N. caninum* antibodies in Australian wild deer, spanning three species (fallow, red and sambar deer). Consequently, we also assessed two commercial cELISA tests validated for detecting *N. caninum* in cattle for their ability to detect *N. caninum* antibodies in serum samples of wild deer. *N. caninum* antibodies were detected in 3.7% (7/189, 95% CI 1.8 – 7.45) of the wild deer serum samples collected in south-eastern Australia (*n* = 189), including 97 fallow deer (*Dama dama*), 14 red deer (*Cervus elaphus*), and 78 sambar deer (*Rusa unicolor*). Overall, our study provides the first detection of *N. caninum* antibodies in wild deer and quantifies deer's potential role in the sylvatic cycle of *N. caninum*.

**Supplementary Information:**

The online version contains supplementary material available at 10.1186/s12917-023-03712-2.

## Background

*Neospora caninum* is an intracellular protozoan parasite with worldwide distribution, affecting domestic and wildlife species [1]. Domestic (dogs) and wild canids (coyotes, wolves, and dingoes) are the definitive hosts of *N. caninum*; they excrete oocysts and become infected by ingesting contaminated herbivores' tissues [2–4]. By contrast, ruminants are intermediate hosts that may become infected through consuming food or water contaminated with oocysts shed by definitive hosts (horizontal transmission) [4, 5]. Vertical or transplacental transmission can occur in intermediate hosts and represents the major mode of *N. caninum* transmission in cattle [2]. Neosporosis is the leading cause of spontaneous abortions in cattle worldwide [6], with an annual estimated cost of AU$110 million for the Australian livestock industry, causing a substantial economic and social impact [6–9]. *Neospora* bradyzoites can cause long-term infection within host tissue cysts (chronic infection) and be difficult to detect. Thus, identifying *N. caninum* antibodies is a good indicator of parasite exposure [10].

Detection of *N. caninum* in livestock species and wildlife suggests that the sylvatic cycle, which involves the transmission of a pathogen between wild animals, plays an important role in the epidemiology of this parasite. Antibodies against *N. caninum* have been reported in various wild ruminants worldwide, including deer [2, 4]. The role of deer as a natural intermediate host of *N. caninum* has been reported in Europe and North America [2, 11] and congenital infection was demonstrated in white-tailed deer (*Odocoileus virginianus*) [12]. In addition, white-tailed deer in North America is considered an important intermediate host for this parasite based on a very high antibody prevalence (above 70%) [1, 2, 4].

In cattle and canid species, the indirect immunofluorescent antibody test (IFAT) and the enzyme-linked immunosorbent assay (ELISA) are the most used assays for serologic analysis of *N. caninum* with validated kits available for both host species [4, 13, 14]. However, serological analysis is generally challenging in wild animals because of post-mortem degradation of immunoglobulins (for samples obtained at necropsy), absence of species-specific secondary antibodies or conjugates, and potential cross-reaction with closely related apicomplexan parasites [2, 11, 15]. Despite these limitations, serological studies have provided compelling evidence of *N. caninum* exposure in wildlife species [11].

In Australia, deer were introduced over 150 years ago for hunting purposes. Nowadays, several deer species have established wild populations and coexist with local livestock and wildlife, posing the risk of transmitting endemic and/or introducing novel pathogens [16]. In south-eastern Australia, six deer species have established wild populations with high densities [17], and fallow deer (*Dama dama*) is the most widespread deer species in the country [18]. Wild deer habitats overlap with wild dogs, one of the established definitive hosts of *N. caninum* in Australia [7, 17]. Consequently, deer are likely exposed to the pathogens carried and transmitted by wild dogs, including *N. caninum*. However, the presence of *N. caninum* infections in Australian deer populations is yet to be investigated.

Here, we report the first detection of *N. caninum* antibodies in Australian wild deer, including in three distinct deer species. Moreover, we provide valuable baseline data on antibody profiles in fallow deer, red deer and sambar deer, quantifying the potential role of wild deer in the sylvatic cycle of *N. caninum*.

## Results

### Comparison of two cELISA kits for the detection of N. caninum antibodies in Australian wild deer

**Fig. 1:**
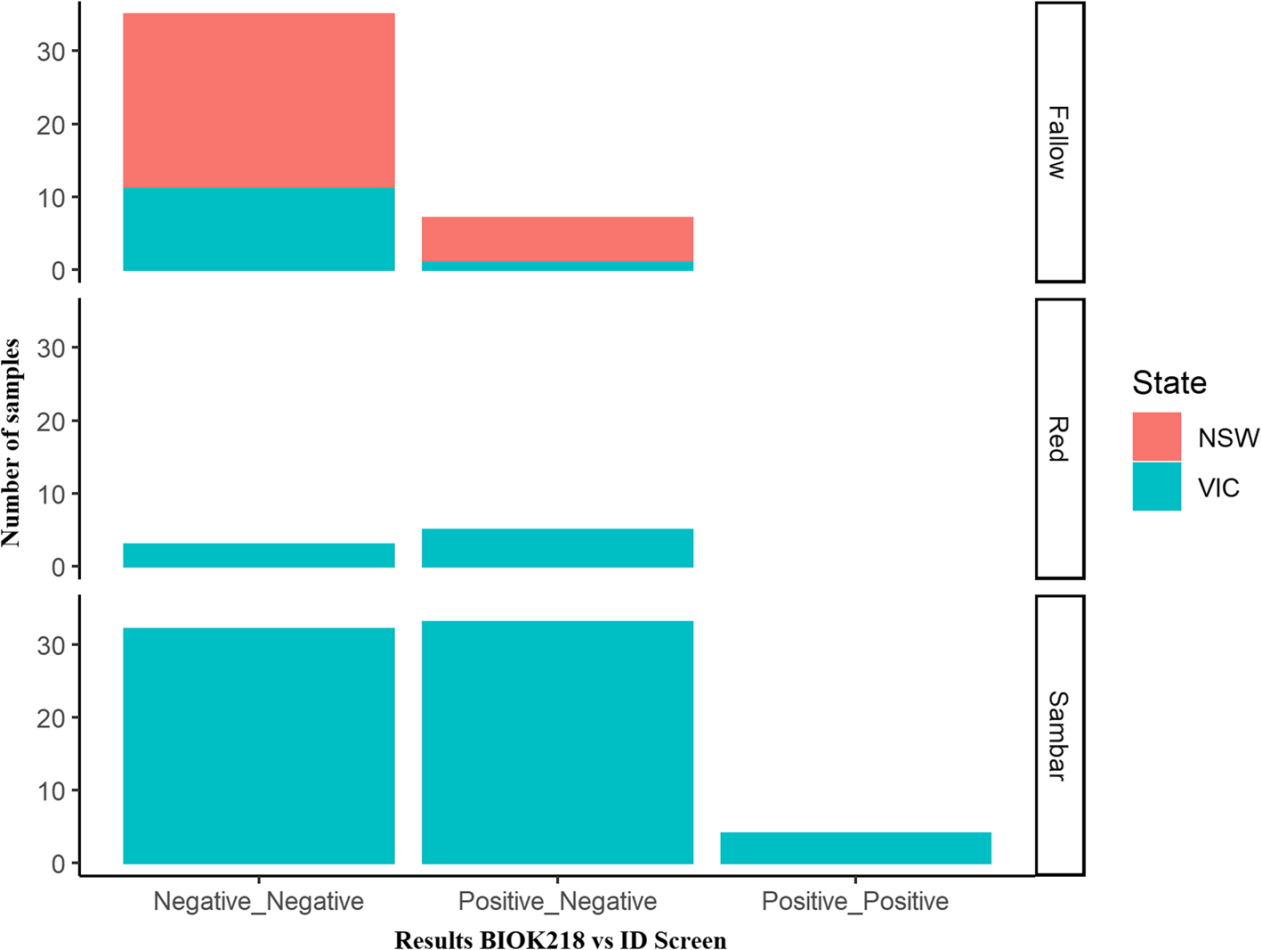
Cross-classified test results obtained by two cELISA tests (BioK218 and ID Screen) to detect the presence of *N. caninum* antibodies in 189 serum samples of Australian wild deer. Samples were collected from fallow, red and sambar deer across the Australian states of New South Wales (NSW) and Victoria (VIC)

A total of 189 wild deer were sampled in south-eastern Australia, encompassing three deer species: 97 fallow deer, 14 red deer, and 78 sambar deer. Of these samples, 119 were used to evaluate the performance of two commercial cELISA kits (BIO K218 and ID Screen). All the positive samples detected with the ID Screen were also positive with the BIO K218 kit. However, many seropositive samples obtained with the BIO K218 kit were negative with the ID Screen kit (Table S1). Cross-classified results demonstrated a large discrepancy between the two kits (Fig. 1).

The sensitivity and specificity posterior probabilities from Bayesian analysis corresponded to the priors except for the specificity of the ELISA BIO K218 (76%, 95% Credibility Interval, CrI: 70–82%) (Table 1). To further investigate the sensitivity of the results to our choice of the prior, we repeated the analysis using a wider prior for the specificity and sensitivity of the ID screen kit (Beta (30,1); 95% probability interval 88.5—97.8%, and the same priors for the Bio K218 in a second analysis), and this did not change our results. Such a strong shift from the priors indicated a strong signal in the data that the specificity of the Bio K218 kit was lower than expected. Moreover, while the median estimates of the correlations between the tests were mildly positive, the 95% credibility interval included zero suggesting inconsistent results between the two tests (Table 1). Therefore, the ID Screen® *Neospora caninum* Competition kit was used to evaluate the remaining 70 wild deer serum samples.

**Table 1:** Bayesian inferences of model parameters based on serological data obtained with two ELISA kits (Bio K218 and ID screen) used to detect *N. caninum* antibodies in wild deer serum samples

Parameter	Mean	SD	2.5%	50%	97.5%
Prevalence (Fallow deer, NSW)	0.087	0.036	0.031	0.082	0.169
Prevalence (Fallow deer, VIC)	0.123	0.050	0.044	0.117	0.238
Prevalence (Red deer, VIC)	0.136	0.055	0.048	0.129	0.262
Prevalence (Sambar deer, VIC)	0.092	0.029	0.044	0.090	0.157
${\rho }_{p}$	0.092	0.078	-0.018	0.080	0.262
${\rho }_{n}$	0.029	0.043	-0.036	0.021	0.132
Se (BIO K218)	0.890	0.025	0.836	0.891	0.933
Sp (BIO K218)	0.763	0.029	0.704	0.764	0.818
Se (ID Screen)	0.993	0.006	0.977	0.995	1.000
Sp (ID Screen)	0.996	0.004	0.986	0.997	1.000

*S.D* Standard deviation, *Se* Sensitivity, *Sp* Specificity, *ρ*() correlation of positives (*p*) or negatives (*n*)

### Seroprevalence of N. caninum in wild deer detected with the ID Screen kit

In addition to the 119 serum deer samples initially tested with both *N. caninum* cELISA kits, the remaining 70 serum samples were tested with the ID Screen® *Neospora caninum* Competition kit (total samples: *n* = 189). The OD values of the 189 sera varied between 0.08 and 1.97. The negative and positive control sera provided mean OD values of 0.06 and 1.39, respectively. Samples tested positive presented OD values ranging from 0.076 to 0.395, resulting in S/N% between 9.1 and 41.5. No doubtful results were obtained in any of the 189 samples tested. Seven samples tested positive for *N. caninum* antibodies using the manufacturer's suggested S/N% cut-off (Table 2). At least one deer sample proved seropositive in each state sampled (Victoria and New South Wales). *N. caninum* antibodies were detected only in fallow deer samples from NSW and sambar deer samples from VIC, although we acknowledge the limited sample size for some of these populations. Overall, *N. caninum* seroprevalence in Victoria across all sampled deer species (fallow, red and sambar deer; *n* = 104) was 4.5%, and the total prevalence in New South Wales (*n* = 85) was 1.2%.

**Table 2:** Seroprevalence of *N. caninum* antibodies detected in wild deer serum samples sampled from south-eastern Australia

Location	Deer species	Total animals	Positive	Prevalence % (95% CI)
New South Wales	Fallow	76	1	1.3 (0.23 – 7.1)
	Red	6	0	0 (0 – 39.0)
	Sambar	3	0	0 (0 – 56.2)
Victoria	Fallow	21	0	0 (0 – 15.5)
	Red	8	0	0 (0 – 32.4)
	Sambar	75	6	8 (3.72 – 16.37)
Total		189	7	3.7 (1.8 – 7.45)

*CI* Confidence interval

## Discussion

Australia's wild deer populations have increased in abundance and distribution during recent decades [17], and the close interaction between deer and livestock is a risk for pathogen transmission [19]. However, little is known about the epidemiology of pathogens that wild deer may transmit to livestock, other domestic animals, or wildlife in Australia. The present study complements our recent investigations on pathogens of wild deer across multiple geographic locations in Australia [20–23]. Detection of *Neospora caninum* antibodies is a key factor in documenting the exposure of wildlife species to the parasite [2]. Exposure to *N. caninum* has been previously reported in wildlife, including deer [2, 8, 15]. However, detecting *N. caninum* antibodies in wildlife species represents a significant challenge due to the lack of validated serological tools and species-specific secondary antibodies [2, 11, 15]. In recent years, the use of competitive ELISA (cELISA) assays has emerged as a trusted approach as they do not require the use of species-specific conjugates; therefore, these tests can theoretically be used to test samples of a different animal species than the one they have been initially designed for [24]. Here, the ability of two *N. caninum* cELISA kits (Bio K218 and ID screen) to detect *N. caninum* antibodies in the serum of Australian wild deer was compared. One hundred and nineteen serum samples were tested with both ELISA kits, and the data was cross-analysed using a Bayesian approach. We made several assumptions with our approach. Firstly, we assumed that information on the sensitivity and specificity of the tests estimated in cattle could be applied to deer. Secondly, we assumed that the tests were performed equally in all deer populations. Lastly, we assumed that the expected prevalence in deer populations would be similar to those in cattle from the same regions (although it should be noted that we used a relatively wide prior range for this parameter). Seroprevalence in Australian cattle was estimated as 10.9% in dairy and 8.7% in beef cattle [4, 6]. Moreover, low seroprevalence was found in sheep [25]. Given the lack of data specific to deer populations and the high degree of (evolutionary) similarity between these ruminant species, we considered these assumptions justifiable.

The Bayesian analysis indicated that the specificity of the BIO K218 kit is lower than 82%, which is much lower than what was expected and reported by the manufacturer for cattle. It is important to understand that since our Bayesian analysis used dependent tests, the parameters were not identifiable unless very narrow ranges were provided in the priors of at least two parameters [26, 27]. For the ID screen test, the 95% probability mass of the prior for the sensitivity and specificity parameters is about 0.03, almost fixing these parameters. According to the manufacturer report, the ID Screen kit provides a sensibility and sensitivity of 100% for testing bovine samples and an excellent correlation with IFAT (Indirect Fluorescent Antibody Test) analysis in water buffalo and canine samples. Hence, given the high performance of this test in cattle, and its recognised reliability in other species, we considered our assumption reasonable. Furthermore, we repeated our analysis with different priors and confirmed the robustness of our results.

The results between the two kits were dramatically discordant (e.g., > 50% prevalence in sambar deer and red deer populations in Victoria based on Bio K218, but supposedly absent in the first and < 6% in the two populations according to the ID screen). Based on our Bayesian analysis, we considered screening the remaining seventy wild deer serum samples with the ID Screen kit more reliable. The overall seroprevalence of *N. caninum* in wild deer from south-eastern Australia was 3.7% using 189 samples. The prevalence was relatively low in the populations where *N. caninum* antibodies were detected (fallow deer in NSW and sambar deer in Victoria) (range 1.3 to 8%). *N. caninum* antibodies were not detected in the other four deer populations (red and sambar deer from NSW and fallow and red deer from Victoria). The prevalence estimated with our Bayesian approach with the initial subset of samples tended to be slightly higher, and this is likely to be a consequence of the prior distribution that we used for this parameter because this parameter is essentially estimated from the prior distribution in the absence of information from the data.

To our knowledge, this study represents the first report of the detection of *N. caninum* antibodies in sambar deer and Australian wild deer. Similar seroprevalence was detected in fallow deer from Europe; 1.4% in the Czech Republic [28] and 2.9% in Poland [29]. In addition, Bartova et al*.* [28] employed cELISA and IFAT as diagnostic tests, founding higher sensitivity in cELISA. Although IFAT is a well-established technique for identifying *N. caninum* exposure, most studies in wildlife, including deer, rely on competitive ELISA techniques (cELISA) due to timely results, easy access, and technical simplicity [2, 30].

It is plausible that wild deer can become infected with *N. caninum* following ingestion of food or water contaminated with oocysts excreted by definite hosts such as domestic or wild dogs [3]. Indeed, active shedding of *N. caninum* oocysts in wild dogs (defined here as dingoes, feral domestic dogs and their hybrids) [31], as well as the report of antibodies in domestic dogs [32], have been confirmed in Australia. Given the presence of competent definite hosts (both domestic and wild dogs) in the sampling area of this study, we conclude that wild deer contribute to the sylvatic cycle of *N. caninum* in the eastern regions of Australia. However, the low parasite seroprevalence established in the present study likely reflects that deer have low contact levels with *N. caninum* contaminated food or water sources.

## Conclusion

In summary, our results indicate that wild deer are exposed to *N. caninum* infection in Australia. This fact could have important implications for maintaining the *N. caninum* sylvatic cycle, especially considering recent increases in densities and distributions of wild deer populations in Australia. Moreover, our study extends the host range for *N. caninum* in Australia and provides useful information for developing future control measures.

## Methods

### Wild deer serum samples

Wild deer serum samples were collected opportunistically during field necropsies conducted in south-eastern Australia between April 2018 and October 2020 in Victoria (VIC) and between August 2019 and June 2021 in New South Wales (NSW). A total of one hundred and eighty-nine serum samples were obtained from wild deer comprising 97 fallow deer (*Dama dama*), 78 sambar deer (*Rusa unicolor*), and 14 red deer (*Cervus elaphus*). Blood was drawn from the jugular vein, the heart or the thoracic cavity and collected in sterile tubes (Becton Dickinson, Franklin Lakes, NJ, USA). Collection tubes were immediately refrigerated and transported to the Molecular Parasitology Laboratory at La Trobe University. Samples were centrifuged at 2,000 × g for 10 min, and serum samples were stored at − 80 °C until analysis.

### Detection of N. caninum antibodies by ELISA test

Two competitive ELISA (cELISA) tests commercially available in Australia were selected for this study: BIO K218—Monoscreen Ab ELISA *Neospora caninum* (Bio-X Diagnostics, Belgique) and ID Screen® *Neospora caninum* Competition kit (IDVET, France). An initial subset of 119 wild deer serum samples was tested with both kits following the manufacturer's instructions. The remaining 70 samples were screened using only the ID screen test based on the results of our analysis (see below). Negative (nc) and positive (pc) controls provided by the manufacturers were included in each ELISA plate, and the samples' absorbance was measured at 450 nm.

In the case of the ELISA BIO K218 kit, the test results were expressed as % inhibition (%INH) = [(OD nc – OD sample)/OD nc] × 100. Serum samples with %INH equal to or higher than 33% were considered positive, while samples with %INH values lower than 33% were considered negative.

In the case of the ELISA ID Screen kit, results were expressed as follows: % sample/negative control (S/N%) = (OD sample/OD negative control) × 100%. Serum samples with S/N% equal to or lower than 50% were considered positive. If the S/N ratio was greater than 60%, the sample was considered negative, while samples with 50% < S/N% ≤ 60% were considered doubtful.

### Statistical analysis

Results from an initial subset of 119 samples screened with both ELISA kits (BIO K218 and ID Screen) and partitioned into four populations (fallow deer, red deer and sambar deer from VIC, and fallow deer from NSW) were analysed using a Bayesian framework. We used the approach developed by Dendekuri and Joseph [26], where the results from the two tests are drawn from a multinomial distribution whose probabilities are a function of the prevalence, the sensitivity and specificity of each test, and the covariance of the two tests. For each serological test, we used sensitivity and specificity from cattle provided by the manufacturers to set the prior for these parameters (Table 3). In the absence of data on seroprevalence for *Neospora* in wild deer populations in Australia, we used published *Neospora* prevalence in cattle from the same geographical areas [4, 33] to guide the selection of a realistic prior for the prevalence parameter (Table 3). While this model has identifiability issues if all parameters have a wide prior, it should be noted that, based on the manufacturer's data, the 95% probability mass for the sensitivity and specificity of the ID screen test is less than 0.1, which should allow the estimation of the other parameters in the model [27]. Lastly, we selected a uniform prior on the covariance parameters with boundaries equal to (Se[Bio K218]-1)(1-Se[ID screen]); min(Se[Bio K218], Se[ID screen])- Se[Bio K218] × Se[ID screen] and (Sp[Bio K218]-1)(1-Sp[ID screen]); min(Sp[Bio K218], Sp[ID screen])-Sp[Bio K218] × Sp[ID screen], where Se[X] and Sp[X] are the sensitivity and the specificity for the test X as recommended by Branscum and colleagues [27]. We then computed the correlations for the positives (*p*) and the negatives (*n*) between the two tests as:

**Table 3:** Prior distributions used in the Bayesian model applied in this study. The model was developed by Dendekuri and Joseph (2001) [26]. Se: sensitivity, Sp: specificity

Parameter	Prior	2.5%	50%	97.5%
Se (BIO K218)	Beta (135,17)	0.84	0.90	0.93
Sp (BIO K218)	Beta (92,5)	0.90	0.95	0.98
Se (ID Screen)	Beta (150,1)	0.98	0.99	0.99
Sp (ID Screen)	Beta (160,1)	0.98	0.99	1.000
Prevalence	Beta (5,24)	0.06	0.17	0.33

$${\rho }_{p}={}^{COVp}\!\left/ \!{}_{\sqrt{\mathrm{Se}[\mathrm{Bio\;K}218](1-\mathrm{Se}\left[\mathrm{Bio\;K}218\right])\mathrm{Se}[\mathrm{ID\;screen}](1-\mathrm{Se}\left[\mathrm{ID\;screen}\right])}}\right.$$

$${\rho }_{n}={}^{COVn}\!\left/ \!{}_{\sqrt{\mathrm{Sp}[\mathrm{Bio\;K}218](1-\mathrm{Sp}\left[\mathrm{Bio\;K}218\right])\mathrm{Sp}[\mathrm{ID\;screen}](1-\mathrm{Sp}\left[\mathrm{ID\;screen}\right])}}\right.$$

We fitted this model to the data using JAGS 4.3.0 [34] run from R 4.0.5 [35] with the R package jagsUI [36]. Four Markov Chain Monte Carlo were run for 100,000 iterations, and the first 5,000 iterations were discarded as burn-in. The estimated sample size was a minimum of 15,000 for each parameter, and the German-Rubin statistic [37] with a threshold of 1.1 was considered to confirm convergence. The final *N. caninum* seroprevalence was calculated based on the proportion of seropositive results among the 189 deer serum samples tested and is presented with a 95% confidence interval (CI), calculated using the Wilson score interval (www.epitools.ausvet.com.au).

### Supplementary Information

**Additional file 1: Table S1. **Characteristics, distribution and results of all the wild deer serum samples (*n*=189) tested in the present study. A) Subset of 119 samples tested with the cELISA kits Bio K218 and ID Screen to assess diagnostic test efficacy. B)  Remaining 70 samples tested only with the ID Screen kit to calculate the overall N. caninum seroprevalence. %INH = % inhibition, S/N% = % sample/negative control.

## Data Availability

All data generated or analysed during this study are included in this published article and its supplementary information files.
