# Supplementary material for: Evidence of Australian wild deer exposure to N. caninum infection and potential implications for the maintenance of N. caninum sylvatic cycle
Source: BMC Vet Res. 2023 Sep 13;19:153. doi: 10.1186/s12917-023-03712-2 (PMC10498561; doi:10.1186/s12917-023-03712-2)
Supplement: Supplementary file 1 — Additional file 1: Table S1. Characteristics, distribution and results of all the wild deer serum samples (n=189) tested in the present study. A) Subset of 119 samples tested with the cELISA kits Bio K218 and ID Screen to assess diagnostic test efficacy. B) Remaining 70 samples tested only with the ID Screen kit to calculate the overall N. caninum seroprevalence. %INH = % inhibition, S/N% = % sample/negative control. [file 12917_2023_3712_MOESM1_ESM.docx]

**Supplementary Material**

**Table S1.** Characteristics, distribution and results of all the wild deer serum samples (n=189) tested in the present study. A) Subset of 119 samples tested with the cELISA kits Bio K218 and ID Screen to assess diagnostic test efficacy. B) Remaining 70 samples tested only with the ID Screen kit to calculate the overall *N. caninum* seroprevalence. %INH = % inhibition, S/N% = % sample/negative control.

**Table S1**

**A)**

| **Host ID** | **Deer species** | **Location** | **Bio K218** | | **ID Screen** | |
| --- | --- | --- | --- | --- | --- | --- |
|  |  |  | **%INH** | **Result** | **S/N%** | **Result** |
| VIC001 | Sambar | Victoria | 49.58 | Positive | 134.13 | Negative |
| VIC002 | Sambar | Victoria | 94.85 | Positive | 16.11 | Positive |
| VIC003 | Sambar | Victoria | 94.02 | Positive | 134.13 | Negative |
| VIC004 | Sambar | Victoria | 54.91 | Positive | 128.88 | Negative |
| VIC005 | Sambar | Victoria | 37.82 | Positive | 133.53 | Negative |
| VIC006 | Sambar | Victoria | 59.58 | Positive | 131.86 | Negative |
| VIC007 | Sambar | Victoria | 47.52 | Positive | 139.50 | Negative |
| VIC008 | Sambar | Victoria | 50.97 | Positive | 123.27 | Negative |
| VIC009 | Sambar | Victoria | 34.42 | Positive | 109.19 | Negative |
| VIC010 | Sambar | Victoria | 39.09 | Positive | 134.73 | Negative |
| VIC011 | Sambar | Victoria | 39.27 | Positive | 150.60 | Negative |
| VIC012 | Sambar | Victoria | 23.09 | Negative | 125.06 | Negative |
| VIC013 | Sambar | Victoria | 22.93 | Negative | 129.59 | Negative |
| VIC014 | Sambar | Victoria | 34.12 | Positive | 154.77 | Negative |
| VIC015 | Sambar | Victoria | 32.20 | Negative | 129.83 | Negative |
| VIC016 | Sambar | Victoria | 25.29 | Negative | 128.76 | Negative |
| VIC017 | Sambar | Victoria | 77.45 | Positive | 9.07 | Positive |
| VIC018 | Sambar | Victoria | 30.17 | Negative | 143.20 | Negative |
| VIC019 | Sambar | Victoria | 12.45 | Negative | 144.51 | Negative |
| VIC020 | Sambar | Victoria | 25.12 | Negative | 118.74 | Negative |
| VIC021 | Sambar | Victoria | 43.06 | Positive | 125.71 | Negative |
| VIC022 | Sambar | Victoria | 37.25 | Positive | 126.39 | Negative |
| VIC023 | Sambar | Victoria | 26.55 | Negative | 116.94 | Negative |
| VIC024 | Sambar | Victoria | 16.68 | Negative | 122.79 | Negative |
| VIC025 | Sambar | Victoria | 14.48 | Negative | 104.18 | Negative |
| VIC026 | Sambar | Victoria | 85.21 | Positive | 123.15 | Negative |
| VIC027 | Sambar | Victoria | 10.73 | Negative | 138.90 | Negative |
| VIC028 | Sambar | Victoria | 19.82 | Negative | 137.71 | Negative |
| VIC029 | Sambar | Victoria | 92.42 | Positive | 115.87 | Negative |
| VIC030 | Sambar | Victoria | 65.39 | Positive | 120.53 | Negative |
| VIC031 | Sambar | Victoria | 6.36 | Negative | 88.31 | Negative |
| VIC032 | Sambar | Victoria | 9.43 | Negative | 115.97 | Negative |
| VIC033 | Sambar | Victoria | 18.87 | Negative | 110.27 | Negative |
| VIC034 | Sambar | Victoria | 10.75 | Negative | 137.11 | Negative |
| VIC035 | Sambar | Victoria | 40.85 | Positive | 101.72 | Negative |
| VIC036 | Sambar | Victoria | 48.06 | Positive | 133.51 | Negative |
| VIC037 | Sambar | Victoria | 44.42 | Positive | 121.29 | Negative |
| VIC038 | Sambar | Victoria | 21.76 | Negative | 121.48 | Negative |
| VIC039 | Sambar | Victoria | 33.33 | Positive | 108.32 | Negative |
| VIC040 | Sambar | Victoria | 30.85 | Negative | 112.97 | Negative |
| VIC041 | Sambar | Victoria | 44.42 | Positive | 116.27 | Negative |
| VIC042 | Sambar | Victoria | 41.70 | Positive | 118.82 | Negative |
| VIC043 | Fallow | Victoria | 32.04 | Negative | 109.43 | Negative |
| VIC044 | Fallow | Victoria | 60.39 | Positive | 116.11 | Negative |
| VIC045 | Sambar | Victoria | 39.50 | Positive | 101.87 | Negative |
| VIC046 | Sambar | Victoria | 36.81 | Positive | 109.60 | Negative |
| VIC047 | Sambar | Victoria | 72.68 | Positive | 167.54 | Negative |
| VIC048 | Sambar | Victoria | 33.08 | Positive | 131.62 | Negative |
| VIC049 | Sambar | Victoria | 60.34 | Positive | 34.13 | Positive |
| VIC050 | Sambar | Victoria | 68.84 | Positive | 41.53 | Positive |
| VIC051 | Sambar | Victoria | 42.57 | Positive | 127.74 | Negative |
| VIC052 | Sambar | Victoria | 35.93 | Positive | 120.61 | Negative |
| VIC053 | Red | Victoria | 44.82 | Positive | 111.54 | Negative |
| VIC054 | Red | Victoria | 45.20 | Positive | 106.45 | Negative |
| VIC055 | Red | Victoria | 41.52 | Positive | 100.37 | Negative |
| VIC056 | Red | Victoria | 40.15 | Positive | 129.39 | Negative |
| VIC057 | Sambar | Victoria | 22.27 | Negative | 115.59 | Negative |
| VIC058 | Sambar | Victoria | 35.98 | Positive | 111.62 | Negative |
| VIC059 | Sambar | Victoria | 37.74 | Positive | 115.22 | Negative |
| VIC060 | Red | Victoria | 40.04 | Positive | 89.96 | Negative |
| VIC061 | Red | Victoria | 25.73 | Negative | 110.72 | Negative |
| VIC062 | Sambar | Victoria | 25.18 | Negative | 120.09 | Negative |
| VIC063 | Sambar | Victoria | 33.02 | Positive | 131.98 | Negative |
| VIC064 | Sambar | Victoria | 7.62 | Negative | 124.34 | Negative |
| VIC065 | Sambar | Victoria | 14.26 | Negative | 120.61 | Negative |
| VIC066 | Sambar | Victoria | 30.12 | Negative | 117.02 | Negative |
| VIC067 | Sambar | Victoria | 26.77 | Negative | 116.27 | Negative |
| VIC068 | Sambar | Victoria | 26.77 | Negative | 110.12 | Negative |
| VIC069 | Sambar | Victoria | 33.46 | Positive | 131.15 | Negative |
| VIC070 | Red | Victoria | 28.85 | Negative | 116.27 | Negative |
| VIC071 | Red | Victoria | 28.85 | Negative | 101.20 | Negative |
| VIC072 | Fallow | Victoria | 8.56 | Negative | 97.90 | Negative |
| VIC073 | Sambar | Victoria | 5.27 | Negative | 122.19 | Negative |
| VIC074 | Sambar | Victoria | 19.09 | Negative | 127.66 | Negative |
| VIC075 | Fallow | Victoria | 15.19 | Negative | 105.40 | Negative |
| VIC076 | Fallow | Victoria | 26.88 | Negative | 98.88 | Negative |
| VIC077 | Fallow | Victoria | 5.65 | Negative | 103.07 | Negative |
| VIC078 | Fallow | Victoria | 15.30 | Negative | 114.44 | Negative |
| VIC079 | Sambar | Victoria | 27.21 | Negative | 100.45 | Negative |
| VIC080 | Fallow | Victoria | 21.28 | Negative | 118.82 | Negative |
| VIC081 | Fallow | Victoria | 26.60 | Negative | 103.90 | Negative |
| VIC082 | Fallow | Victoria | 13.22 | Negative | 115.44 | Negative |
| VIC083 | Fallow | Victoria | 16.90 | Negative | 117.17 | Negative |
| VIC084 | Fallow | Victoria | 23.26 | Negative | 108.02 | Negative |
| VIC085 | Sambar | Victoria | 41.80 | Positive | 133.05 | Negative |
| VIC086 | Sambar | Victoria | 23.53 | Negative | 110.34 | Negative |
| VIC087 | Sambar | Victoria | 16.46 | Negative | 109.90 | Negative |
| VIC088 | Sambar | Victoria | 28.36 | Negative | 118.14 | Negative |
| VIC089 | Sambar | Victoria | 20.44 | Negative | 112.59 | Negative |
| NSW300 | Fallow | New South Wales | 36.89 | Positive | 99.16 | Negative |
| NSW301 | Fallow | New South Wales | 32.46 | Negative | 109.66 | Negative |
| NSW302 | Fallow | New South Wales | 34.75 | Positive | 117.20 | Negative |
| NSW303 | Fallow | New South Wales | 29.06 | Negative | 103.92 | Negative |
| NSW304 | Fallow | New South Wales | 35.45 | Positive | 106.16 | Negative |
| NSW305 | Fallow | New South Wales | 30.92 | Negative | 108.63 | Negative |
| NSW306 | Fallow | New South Wales | 30.36 | Negative | 104.28 | Negative |
| NSW307 | Fallow | New South Wales | 20.29 | Negative | 98.25 | Negative |
| NSW308 | Fallow | New South Wales | 39.65 | Positive | 105.61 | Negative |
| NSW309 | Fallow | New South Wales | 26.21 | Negative | 105.01 | Negative |
| NSW310 | Fallow | New South Wales | 35.31 | Positive | 115.87 | Negative |
| NSW311 | Fallow | New South Wales | 31.16 | Negative | 99.09 | Negative |
| NSW312 | Fallow | New South Wales | 38.81 | Positive | 103.98 | Negative |
| NSW313 | Fallow | New South Wales | 28.96 | Negative | 109.05 | Negative |
| NSW314 | Fallow | New South Wales | 28.36 | Negative | 84.19 | Negative |
| NSW315 | Fallow | New South Wales | 32.28 | Negative | 97.71 | Negative |
| NSW316 | Fallow | New South Wales | 25.14 | Negative | 92.09 | Negative |
| NSW317 | Fallow | New South Wales | 32.74 | Negative | 92.28 | Negative |
| NSW318 | Fallow | New South Wales | 26.54 | Negative | 105.25 | Negative |
| NSW319 | Fallow | New South Wales | 21.22 | Negative | 107.60 | Negative |
| NSW320 | Fallow | New South Wales | 24.49 | Negative | 95.29 | Negative |
| NSW321 | Fallow | New South Wales | 17.91 | Negative | 111.29 | Negative |
| NSW322 | Fallow | New South Wales | 21.46 | Negative | 97.65 | Negative |
| NSW323 | Fallow | New South Wales | 22.76 | Negative | 94.93 | Negative |
| NSW330 | Fallow | New South Wales | 16.70 | Negative | 91.07 | Negative |
| NSW331 | Fallow | New South Wales | 13.43 | Negative | 94.51 | Negative |
| NSW332 | Fallow | New South Wales | 24.77 | Negative | 96.38 | Negative |
| NSW333 | Fallow | New South Wales | 13.15 | Negative | 99.03 | Negative |
| NSW334 | Fallow | New South Wales | 11.57 | Negative | 97.40 | Negative |
| NSW335 | Fallow | New South Wales | 16.65 | Negative | 110.44 | Negative |

**B)**

| **Host ID** | **Deer species** | **Location** | **ID Screen** | |
| --- | --- | --- | --- | --- |
|  |  |  | **S/N%** | **Result** |
| NSW324 | Fallow | New South Wales | 111.01 | Negative |
| NSW325 | Fallow | New South Wales | 122.12 | Negative |
| NSW326 | Fallow | New South Wales | 106.21 | Negative |
| NSW327 | Fallow | New South Wales | 122.29 | Negative |
| NSW328 | Fallow | New South Wales | 121.77 | Negative |
| NSW329 | Fallow | New South Wales | 121.77 | Negative |
| NSW336 | Fallow | New South Wales | 143.47 | Negative |
| NSW337 | Fallow | New South Wales | 143.47 | Negative |
| NSW338 | Fallow | New South Wales | 140.51 | Negative |
| NSW339 | Fallow | New South Wales | 134.60 | Negative |
| NSW340 | Fallow | New South Wales | 140.34 | Negative |
| NSW341 | Fallow | New South Wales | 128.95 | Negative |
| NSW342 | Fallow | New South Wales | 127.43 | Negative |
| NSW343 | Fallow | New South Wales | 131.14 | Negative |
| NSW344 | Fallow | New South Wales | 120.01 | Negative |
| NSW345 | Fallow | New South Wales | 125.82 | Negative |
| NSW346 | Fallow | New South Wales | 139.66 | Negative |
| NSW347 | Fallow | New South Wales | 135.36 | Negative |
| NSW348 | Fallow | New South Wales | 142.45 | Negative |
| NSW349 | Fallow | New South Wales | 131.56 | Negative |
| NSW350 | Fallow | New South Wales | 115.61 | Negative |
| NSW351 | Fallow | New South Wales | 137.47 | Negative |
| NSW352 | Fallow | New South Wales | 33.33 | Positive |
| NSW353 | Fallow | New South Wales | 124.47 | Negative |
| NSW354 | Fallow | New South Wales | 133.00 | Negative |
| NSW355 | Fallow | New South Wales | 130.38 | Negative |
| NSW356 | Fallow | New South Wales | 140.84 | Negative |
| NSW357 | Fallow | New South Wales | 128.61 | Negative |
| NSW358 | Fallow | New South Wales | 133.42 | Negative |
| NSW359 | Fallow | New South Wales | 137.30 | Negative |
| NSW360 | Fallow | New South Wales | 118.14 | Negative |
| NSW361 | Fallow | New South Wales | 134.48 | Negative |
| NSW362 | Fallow | New South Wales | 131.03 | Negative |
| NSW363 | Fallow | New South Wales | 131.03 | Negative |
| NSW364 | Fallow | New South Wales | 131.56 | Negative |
| NSW365 | Fallow | New South Wales | 131.56 | Negative |
| NSW366 | Fallow | New South Wales | 137.20 | Negative |
| NSW367 | Sambar | New South Wales | 137.20 | Negative |
| NSW368 | Sambar | New South Wales | 118.70 | Negative |
| NSW369 | Fallow | New South Wales | 118.70 | Negative |
| NSW370 | Fallow | New South Wales | 114.94 | Negative |
| NSW371 | Fallow | New South Wales | 114.94 | Negative |
| NSW372 | Fallow | New South Wales | 96.55 | Negative |
| NSW373 | Fallow | New South Wales | 96.55 | Negative |
| NSW374 | Fallow | New South Wales | 127.90 | Negative |
| NSW375 | Fallow | New South Wales | 127.90 | Negative |
| NSW376 | Fallow | New South Wales | 108.88 | Negative |
| NSW377 | Fallow | New South Wales | 108.88 | Negative |
| NSW378 | Red | New South Wales | 163.85 | Negative |
| NSW379 | Red | New South Wales | 163.85 | Negative |
| NSW380 | Red | New South Wales | 126.40 | Negative |
| NSW381 | Red | New South Wales | 121.85 | Negative |
| NSW382 | Red | New South Wales | 114.60 | Negative |
| NSW383 | Sambar | New South Wales | 113.37 | Negative |
| NSW384 | Red | New South Wales | 116.43 | Negative |
| VIC90 | Sambar | Victoria | 138.14 | Negative |
| VIC91 | Sambar | Victoria | 165.99 | Negative |
| VIC92 | Sambar | Victoria | 133.67 | Negative |
| VIC93 | Fallow | Victoria | 137.72 | Negative |
| VIC94 | Fallow | Victoria | 135.11 | Negative |
| VIC95 | Fallow | Victoria | 117.76 | Negative |
| VIC96 | Fallow | Victoria | 129.36 | Negative |
| VIC97 | Fallow | Victoria | 129.36 | Negative |
| VIC98 | Fallow | Victoria | 128.42 | Negative |
| VIC99 | Sambar | Victoria | 128.42 | Negative |
| VIC100 | Fallow | Victoria | 127.48 | Negative |
| VIC101 | Fallow | Victoria | 127.48 | Negative |
| VIC102 | Fallow | Victoria | 123.25 | Negative |
| VIC103 | Sambar | Victoria | 26.75 | Positive |
| VIC104 | Sambar | Victoria | 17.14 | Positive |
